# Genome-wide analysis of DNA methylation in hypothalamus and ovary of *Capra hircus*

**DOI:** 10.1186/s12864-017-3866-4

**Published:** 2017-06-23

**Authors:** Stefano Frattini, Emanuele Capra, Barbara Lazzari, Stephanie D. McKay, Beatrice Coizet, Andrea Talenti, Debora Groppetti, Pietro Riccaboni, Alessandro Pecile, Stefania Chessa, Bianca Castiglioni, John L. Williams, Giulio Pagnacco, Alessandra Stella, Paola Crepaldi

**Affiliations:** 1 Department of Veterinary Science, University of Milan, Milan, Italy; 2 Institute of Agricultural Biology and Biotechnology, National Research Council UOS of Lodi, Lodi, Italy; 3 PTP Science Park, Lodi, Italy; 4 Department of Animal & Veterinary Sciences, University of Vermont, Burlington, VT USA; 5 The Davies Research Centre, School of Animal and Veterinary Sciences, University of Adelaide, Roseworthy, 5371 Australia

**Keywords:** Goat, DNA methylation, Epigenetic, MBD-seq, Hypothalamus, Ovary, RNA-seq

## Abstract

**Background:**

DNA methylation is a frequently studied epigenetic modification due to its role in regulating gene expression and hence in biological processes and in determining phenotypic plasticity in organisms. Rudimentary DNA methylation patterns for some livestock species are publically available: among these, goat methylome deserves to be further explored.

**Results:**

Genome-wide DNA methylation maps of the hypothalamus and ovary from Saanen goats were generated using Methyl-CpG binding domain protein sequencing (MBD-seq). Analysis of DNA methylation patterns indicate that the majority of methylation peaks found within genes are located gene body regions, for both organs. Analysis of the distribution of methylated sites per chromosome showed that chromosome X had the lowest number of methylation peaks. The X chromosome has one of the highest percentages of methylated CpG islands in both organs, and approximately 50% of the CpG islands in the goat epigenome are methylated in hypothalamus and ovary. Organ-specific Differentially Methylated Genes (DMGs) were correlated with the expression levels.

**Conclusions:**

The comparison between transcriptome and methylome in hypothalamus and ovary showed that a higher level of methylation is not accompanied by a higher gene suppression. The genome-wide DNA methylation map for two goat organs produced here is a valuable starting point for studying the involvement of epigenetic modifications in regulating goat reproduction performance.

**Electronic supplementary material:**

The online version of this article (doi:10.1186/s12864-017-3866-4) contains supplementary material, which is available to authorized users.

## Background

DNA methylation has a key role in regulating gene expression. The addition of a methyl group to the 5-carbon position of a cytosine is carried out by a class of enzymes known as DNA-methyltransferases (DNMT) using S-adenosyl-methionine as the methyl donor. DNMT1 maintains DNA methylation during DNA replication while DNMT3a and DNMT3b are responsible for de novo methylation [1]. The connection between methylation and gene expression is intricate, with high levels of gene expression frequently associated with low levels of methylation in the promoter region of genes [2]. Methylation within genes has been hypothesized to influence gene expression levels by reducing the rate of transcriptional elongation, however, the cellular mechanism linking gene-body methylation and gene activity remains unclear [3–5]. DNA methylation patterns can be inherited and influenced by the environment, diet and aging [6]. It has been also shown that methylation plays a key role in X-chromosome inactivation [7], differentiation and development of tissues and imprinting of genes [8, 9], while aberrant DNA methylation is implicated in many types of disease, including cancer [4, 10]. In mammals, methylation is commonly found at the 5-carbon position of cytosines and is predominantly observed at CpG dinucleotides and especially in GC-rich regions called CpG islands (CGIs). CGI are often associated transcription star site of genes, and CGI methylation is a potential gene marker [9]. CpG islands represent an important gene feature that are used in gene prediction, gene feature analysis, and epigenetic studies [11]. A prerequisite for understanding the function of DNA methylation is the distribution in the genome. Methyl-CpG binding domain protein sequencing (MBD-seq) is a cost efficient method to investigate locations of genome-wide methylation with high efficiency and resolution [12]. Genome-wide DNA methylation studies of many livestock species have been recently reported [13–17]. These studies used several methods to explore tissue specific methylation associated with economically important traits. Methylomes have been explored in sheep and pig using reduced representation bisulfite sequencing (RRBS) while MeDIP-seq has been used for studies in horse, cow and chicken. Epigenetic modifications have been associated with phenotypic variation in livestock species [18]. Phenotypic variation in economically important traits, such as lipid synthesis, milk production, growth and development, that are not explained by genetics could be influenced by epigenetic factors, such as the wide spectrum of methylation levels and patterns seen in livestock [19].

*Capra hircus* is an economically important livestock species that is globally distributed, especially in developing countries. It is an indispensable part in the animal fiber, meat and milk industries [20]. The world’s goat population was estimated at approximately 1 billion in 2012, with a 12% population increase since 2005 [21]. DNA methylation of the goat genome has yet to be studied. The present study analyzed DNA methylation patterns of two key organs in the hypothalamic-pituitary-gonadal (HPG) axis: hypothalamus and ovary. This axis undergoes a number of changes throughout the life of an individual and is responsible for development, puberty, maturation, and senescence of reproductive systems [22]. Epigenetic modifications play a key role in the complex regulation of this system [9, 23]. The present research compares and contrasts the methylome of ovary and hypothalamus of *Capra hircus* to investigate the impact of DNA methylation on gene expression.

## Results

### Mapping of DNA methylation in Saanen goats

Methyl binding domain sequencing was performed on two reproduction-associated organs (hypothalamus and ovary) of three Saanen goats. Between 23 and 37 million raw sequencing reads were generated from each sample. Following quality control and trimming, approximately 30 million reads per sample remained of which 98% were mapped to the goat reference genome (CHIR_1.0 GenBank assembly accession: GCA_000317765.1) and subsequently analyzed (Table 1). A clonal fraction of sequence reads was removed from each sample, resulting in 65.7% (of the total mapped reads) uniquely mapped reads for the hypothalamus and 71.1% for the ovary, with a high quality read alignment against the goat reference genome version 1.0 (2.64 Gb). Methylated regions (MRs), identified as peaks, were detected among uniquely mapped reads. A total of 382,850 methylation peaks were found in the hypothalamus and 413,010 in the ovary. Peaks were distributed across all chromosomes and covered approximately 28% of the goat genome in the hypothalamus and 32% in the ovary (Table 1).

**Table 1:** Data generated by MBD-seq

Sample	Organ	Total number of raw sequence reads	Percentage of mapped reads in total reads (%)	Percentage of genome coverage (%)
Goat 1	Hypothalamus	31,842,637	98.16	29.17
	Ovary	35,390,803	97.78	35.59
Goat 2	Hypothalamus	23,765,604	98.02	26.75
	Ovary	37,185,292	98.35	40.86
Goat 3	Hypothalamus	27,090,852	98.05	27.96
	Ovary	25,704,845	97.89	20.63

Genome coverage as the percentage of bases mapped by genome-wide reads

### Chromosomal distribution of highly methylated region

**Fig. 1:**
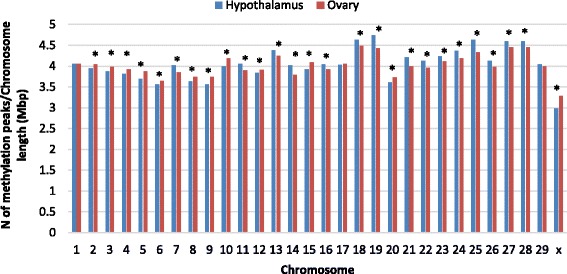
Ratio of the distribution of methylation peaks in goat hypothalamus and ovary by Chromosome. The number of methylation peaks per chromosome on the y axis was corrected by chromosome length in Mbp and by the total number of peaks per organ. For a clearer graphical representation, every ratio was multiplied by 10 k. * = significant at *P* > 0.05

Of the 30 chromosomes in the goat genome, 27 chromosomes had a significantly (*P* < 0.05) different number of methylation peaks between hypothalamus and ovary (Fig. 1). Fifteen chromosomes were significantly more methylated in hypothalamus and twelve chromosomes has more methylation peaks in ovary. Goat chromosome (CHI) X showed the greatest difference in methylation between the two organs (*P* = 4.45E-12), with a higher level of methylation in ovary. Only CHI 1, 17 and 29 did not show significant difference in methylation between the two organs.

### DNA methylation profile across the goat genome

**Fig. 2:**
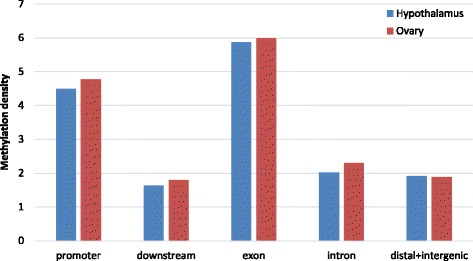
Methylation density in different genomic regions in hypothalamus and ovary. Methylation density within gene regions was calculated by dividing the peak length in the region by the average length (in bp) of the genomic region itself in the genome and by the total number of peaks per organ

**Fig. 3:**
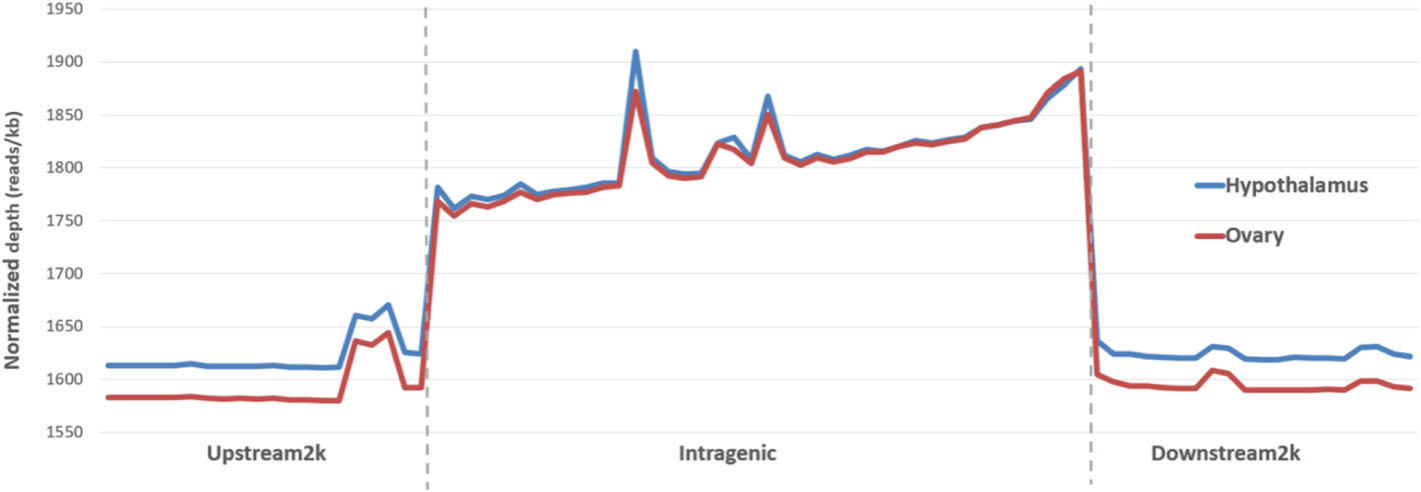
DNA methylation distribution in goat gene region. The DNA methylation profile in gene regions for hypothalamus and ovary shown as the reads aligned with unique loci in the genome. Gene regions including 2 kb upstream of the CDS start, the gene body from CDS start to CDS end, and 2 kb downstream of the CDS end. In the upstream and downstream 2 kb regions, the regions were split into 20 non-overlapping windows, and the average alignment depth was calculated for each window. In the gene body, each gene was split into 40 equal windows, and the average alignment depth was calculated for each window. The y-axis is the average of the normalized depth for each window

Methylation peaks identified in the two organs were connected to five genomic regions, according to where they occurred with respect to the CHIR1.0 reference annotation. The highest density of MRs was observed in exons and promoters for both organs. Conversely, MR density decreases in introns, downstream, distal and intergenic regions (Fig. 2). Methylation distribution across genes and gene boundaries (2 kb upstream region, intragenic and 2 kb downstream region) was investigated. The DNA methylation level was found to increase sharply at the start of the coding sequence (CDS) and continued to increase until the end of the CDS. Subsequently, the DNA methylation level drops dramatically and remains steadily low within the 3′ boundary (Fig. 3). Some differences were observed between the two organs, with the hypothalamus showing a higher methylation level within the upstream and downstream regions with respect to the ovary.

### Distribution of DNA methylation in CGIs

**Fig. 4:**
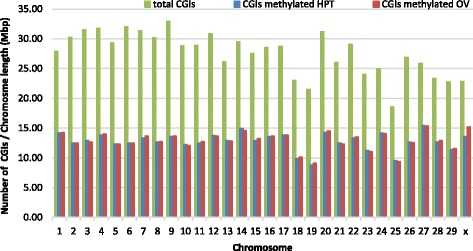
Genomic distribution of total and methylated CpG islands. Green bars show the total number of CGIs identified in the goat genome by chromosome; *blue* and *red bars* indicate the number of the methylated CGIs in hypothalamus (HPT) and ovary (OV) respectively. The number of CGIs per chromosome on the y axis is corrected by chromosome length in Mbp. The thirteen CHI showing different methylation level (*P* < 0.05) between the two organs were, in descending order of their statistical significance, X, 3, 14, 7, 10, 11, 21, 23, 5, 25, 15, 12 and 27

To investigate methylated CGIs the goat reference genome version 1.0 was used to identify CGIs. A total of 71,367 CGIs, representing 0.86% of the whole genome, were identified (Additional file 1). Approximately 45.80% (*n* = 32,683) of the CGIs in hypothalamus were found among the MRs and 46.17% (*n* = 32,952) in ovary. Therefore, a slightly higher CGIs methylation level (+0.37%) was observed in ovary compared to hypothalamus (Fig. 4). Of the thirty chromosomes in the *Capra hircus* genome, thirteen show a significant difference in methylation (*P* < 0.05) between hypothalamus and ovary. Among these, four chromosomes (X, 7, 11 and 15) show a higher DNA methylation level in the CGIs in the ovary and the remaining nine chromosomes (3,14,10,21,23,5,25) show higher DNA methylation levels in hypothalamus (Additional file 2). The greatest difference in CGIs methylation between the organs was observed on CHI X. *Capra hircus* chromosome 6 had the same number of methylated regions in CGIs in both tissues. Comparing the methylation level found in CGIs to that observed for CpGs, CHI X, which was less methylated considering the global level of DNA methylation, became one of the most methylated within only CGIs. Conversely, for CHI 18 and 19 the most methylated chromosome for both organs, showed two of the lowest level of methylated CGIs.

### Differentially Methylated Regions (DMRs) and Genes (DMGs) between hypothalamus and ovary

The difference in the methylation level between hypothalamus and ovary was measured considering the respective differentially methylated genomic regions. The analysis revealed 4808 differentially methylated regions (DMRs) (False Discovery Rate - FDR < 0.05), among which, 1547 were significantly more methylated in the hypothalamus and 3261 in the ovary. A total of 2651 DMRs were located within the gene body, gathered in 1264 differentially methylated genes (DMGs) in ovary and 456 in hypothalamus. In addition, 74 highly methylated genes were in common between hypothalamus and ovary (with DMRs in gene bodies), for a total of 1646 DMGs identified (Additional file 3).

### Differentially Expressed Genes (DEGs) between hypothalamus and ovary

RNA-seq analysis was performed on the same samples to evaluate the variation of gene expression between the two organs. Approximately 330 million paired end (PE) reads were produced per sample which were assembled onto 13,686 unique genes identified from both organs. A total of 7173 differentially expressed genes (FDR < 0.05) were identified (Additional file 4), of which 2665 had significantly higher expression in the hypothalamus and 4508 in the ovary.

### Comparison between DEGs and DMGs

DMGs and DEGs were compared in order to evaluate possible interactions between gene methylation and expression. Different lists of DEGs were selected based on three different FDR values (5512 DEGs for FDR < 0.01, 2722 for FDR < 0.0001, 1424 for FDR <0.000001) and compared with DMGs list (1646 genes) these comparisons identified 620, 349 and 215 shared genes between DEGs and DMGs, respectively. A simulation test based on random selection of loci (jackknifing) showed that the number of shared genes was higher than random expectation and the significance (*P*-value) was inversely proportional to the FDR values for the thresholds considered (Table 2).

**Table 2:** Intersections and simulations between DEGs and DMGs

DEGs thresholds	n° of DEGs	n° of DMGs (FDR < 0.05)	DEGs ∩ DMGs	100 K simulations^a^ of ∩	± SD	*P*-value
FDR < 0.01	5512	1646	620	496.41	17.67	9.55E-12
FDR < 0.0001	2722	1646	349	245.22	13.78	1.92E-13
FDR < 0.000001	1424	1646	215	128.24	10.41	3.20E-16

^a^100 k simulations refer to the jackknifing test used for the random crossing of DEGs and DMGs

To explore the potential regulatory role of methylation on gene expression, a list of DMGs and DEGs (1646 DMGs with FDR < 0.05 and 2722 DEGs with FDR < 0.0001) were divided accordingly to their level of gene methylation and expression, respectively. Thus, four subsets were obtained (hyper and hypo-methylated genes versus over and under-expressed genes) which were cross checked to obtain lists of shared genes (DEGs ∩ DMGs). In hypothalamus, a negative correlation was found between gene expression and hypo-methylation of 231 genes (*P* = 1.29E-46). There was also a significant negative correlation (*P* = 0.0167) between hyper-methylated genes and gene expression. However, considering positive correlations, hyper-methylated genes were not correlated with overexpressed genes. Conversely, the number of genes in common between hypo-methylated and under-expressed genes was less than half of the 100 k simulation test (42 observed versus 103 expected). In this case, the significant correlation (*P* = 1.97 E-10) suggested an opposite (negative) correlation between hypo-methylated genes and those under-expressed (Table 3).

**Table 3:** Correlation between DMGs and DEGs

	Hypothalamus	Obs. DMGs	Obs. DEGs	DEGs ∩ DMGs	100 k simulation of ∩	±SD	*P*-value
Positive Correlation	Hyper DMGs/over DEGs	455	1138	34	30.46	5.25	0.318
	Hypo DMGs/under DEGs	1263	1583	42	103.44	9.38	1.97E-10
Negative Correlation	Hyper DMGs/under DEGs	455	1583	49	34.89	5.60	0.0167
	Hypo DMGs/over DEGs	1263	1138	231	99.10	9.11	1.29E-46

Hypo and hyper-methylated genes (FDR < 0.05) overlapping with under and overexpressed genes (FDR < 0.001) in the hypothalamus (DEGs ∩ DMGs). A jackknife simulation test with relative *P*-values (observed vs random expectation) was performed for each subgroup

### Analysis of functional categories of DEGs and DMGs

Functional annotation analysis using STRING v10.0 was performed for shared genes between DMGs and DEGs in hypothalamus and ovary in order to investigate pathways with and epigenetic regulation. Biological pathway analysis for hypo-methylated genes that were overexpressed in hypothalamus, identified several pathways characteristic of this organ. 114 significant pathways (FDR < 0.0001) were associated with the hypo-methylated genes, which primarily involved synaptic transmission and neuron morphogenesis and development, with almost 200 gene involved in the pathways. Cellular component analysis of the same genes revealed 44 significant pathways (FDR < 0.0001) affecting synapses, neuron and axons. No significant pathways (FDR < 0.01) were detected for hyper-methylated and overexpressed genes for both biological pathways and cellular component analysis. Considering under-expressed genes in hypothalamus (and consequently over-expressed in ovary), there were no significant pathways for hypo-methylated genes. There were 20 significant biological pathways (FDR < 0.01) and 11 pathways for cellular component (FDR < 0.01) for hyper-methylated genes, mainly associated with extracellular matrix, system and tissue development and collagen processes (Additional file 5).

## Discussion

Considering the methylation density across gene regions, hypothalamus and ovary showed a similar trend: exons and promoters had the highest methylation frequency. This high methylation density in exons has already been observed in horse, rat and human [17, 24, 25]. However, the high density of methylation in promoter regions seen in the present study was unexpected. It is well documented that most promoters have a low level of methylation and subsequently facilitate gene expression [26]. The high level of methylation could reflect the type of tissues studied.

In a recent work, researchers presented the DNA methylation pattern in the hypothalamus of female pubertal goats [27]. Despite their goal was to measure DNA methylation in the same organ across puberty, and not at the same physiological time among organs, there are some analogies with our work. In their comparison between DMGs and DEGs they confirmed the influence of DNA methylation on gene expression, showing a high methylation level in promoter regions. Nevertheless, they observed the highest methylation level in introns, unlike our findings where exons were the gene part with the highest methylation density. This difference could be due by the fact that they did not correct the number of differentially methylated regions by the length of each gene part in the genome. In this way introns are more represented than exons because of their overall length.

Compared with other livestock, goats show similar distribution of DNA methylation across different gene regions [14, 16, 17], e.g. comparing the DNA methylation distribution pattern in goat to that found in bovine placenta and horse [14, 17]. In bovine placenta, the DNA methylation level decreased before the TSS, noticeably increases in intragenic regions, and is constantly low downstream of genes. Methylation density in several tissues in horse, also showed a decrease before the TSS, followed by a gradual increase of methylation in the gene body with an acute decline at the TTS and subsequent plateau. Measures of methylation density in hypothalamus and ovary in goat indicates a slight increase before the CDS start, followed by a gradual increase in the intragenic region which contains scattered areas of increased methylation. After the CDS end the DNA methylation level falls sharply and remains constantly low for all the downstream region in both organs.

Analysis of peaks of methylation per chromosome showed that CHI X has the lowest number of methylation peaks. A possible explanation is that the X chromosome has one of the lowest CpG content within the genome. On the other hand, the X chromosome has one of the highest percentages of methylated CpG islands in both hypothalamus and ovary (59.36% and 66.52% respectively). Despite the lower degree of methylation, strong methylation in CpG islands could suggest a gene regulation of the CHI X. This high level of methylation observed in CGIs may be associated with X-chromosome inactivation [28, 29].

The associations between CGIs DNA methylation and gene expression found here were only partially in accordance with previous findings in mammals [13, 14, 16, 17]. CpG islands have usually been reported to be regions of gene regulation via DNA methylation, most likely through the mechanism of transcriptional repression. These regions in vertebrate genomes are known to be generally unmethylated, in spite of having a high GC content [30]. However, recent findings suggest that a relatively high level of DNA methylation can occur in CGIs [8, 31]. In the present study, CpG islands showed a higher methylation density compared with previous studies in other species. Recent works on horse, cattle and chicken epigenomes found 10 to 20% of methylated CGIs across the genomes [14, 16, 17]. The results in the present work indicate that almost 50% of the CGIs in goat are methylated in hypothalamus and ovary. This may be a result of the enrichment method used [32]. However, in the only paper available in goat species about genome-wide DNA methylation pattern, authors stated that the majority of CGIs observed were hypermethylated [27]. This is consistent with our findings. Recent evidence suggests a more complicated role of DNA methylation than simply inhibitory expression [2]. In the present paper, the correlation between RNA-seq and methyl-seq profiles in the two tissues was more consistent for genes with greater differences in expression.

The comparison of hypo/hyper-methylated with the under/over-expressed genes showed that the methylation of a significant fraction of DMGs was negatively correlated with the expression level, in agreement with the idea that DNA methylation represses gene expression [33]. However, the two organs considered here may exhibit different effects of methylation. A strong negative correlation between hypo-methylation and over-expression was seen in the hypothalamus (*P* = 1.26E-46) while only a slight increase in expression was seen in the ovary (*P* = 0.0167). In contrast with a previous reported study in human [34], there was no positive correlation between gene methylation and expression. In a recent study on bovine somatic tissue, methylation in the upstream 1500-bp regions of TSS showed a negative correlation with gene expression [35]. In our work, we could not find any correlation between TSS methylation and the mRNA expression, probably related to incomplete gene annotation in the goat genome.

Pathway analysis revealed that the highly expressed, low methylated genes in the hypothalamus are involved in brain specific signaling. No correlation with specific pathways and tissue function was observed in the ovary. Thus, an interesting observation that emerge from this study is that a low level of gene methylation in CpGs is linked to a high level of expression in the hypothalamus. This is in contrast to what has been observed elsewhere, with a high level of methylation in gene body being associated with a high expression level or with a mixed trend [2, 35, 36]. Epigenetic regulation in the hypothalamus may be in line with the observation that for slowly dividing and non-dividing cells, such as those in the brain, gene body methylation is not associated with increased gene expression [33]. The comparison of the transcriptomes between hypothalamus and ovary showed that a higher level of methylation is not necessarily accompanied by the suppression of expression. This, confirms that methylation is not constantly associated with gene silencing, and its effect on gene expression regulation could be both positive and negative [5, 34].

## Conclusions

This work presents a global methylation pattern for hypothalamus and ovary in *Capra hircus*. The differences observed at the DNA methylation level in hypothalamus and ovary suggest the tissue or organ-specificity of methylation patterns. The DNA methylation landscape of the *Capra hircus* ovary and hypothalamus methylomes associated with the analysis of the transcriptome has highlighted the complexity of epigenetic regulation. The better understanding of the influence of epigenetics on livestock development, response to complex diseases and production traits under different conditions is likely to aid increased animal productivity and sustainability [18].

## Methods

### Genomic DNA and RNA extraction

Three adult female Saanen goats, aged 43.3 ± 3.2 months (mean ± SD) and weighing 55.0 ± 2.3 kg (mean ± SD), reared on the same farm and at the end of their productive career were sacrificed. Animals were euthanized by IV injection of a 10 mL solution of embutramide, mebezonium iodide and tetracaine hydrochloride (Tanax) under anesthetic condition (Ketamine, 5 mg/kg/IV and Diazepan 1 mg/kg/IV). Biological samples of the hypothalamus and ovaries were collected from each goat. The whole hypothalamus and a homogeneous portion of the ovary were frozen in liquid nitrogen and ground to a fine powder, using mortar and pestle and stored at −80 °C until DNA extraction. Genomic DNA from ovary was isolated using the commercial kit NucleoSpin® Tissue (Macherey-Nagel, Düren, Germany). For hypothalamus, phenol:chloroform genomic DNA extractions were performed. About 50 mg of tissue were resuspended in 300ul of TRIS EDTA lysis buffer (10 mM Tris-HCl, 10 mM EDTA, NaCl 250 m, pH 8), and 15 μl of proteinase K (20 mg/ml) (Sigma-Aldrich., St. Louis, MO, USA) and 15 μl of SDS 10% added then incubated at 56 °C for 2 h. Next, 25 μl of RNase A (20 mg/ml) (Sigma-Aldrich) was added to the suspension and incubated at 56 °C for 30 min. DNA was extracted using an equal volume of 1:1 (*v*/v) phenol:chloroform [37] and precipitated with 1 Vol. of cold Isopropanol. DNA was washed with 70% (*v*/v) cold ethanol, air dried then resuspended in 30 μl of ultrapure water and stored at −20 °C until use. DNA concentration and quality were estimated by PicoGreen® (Thermo Fisher, Waltham, MA USA) and by agarose gel electrophoresis. Total RNA was extracted from each with Trizol (Invitrogen, Carlsbad, CA) and purified by NucleoSpin® miRNA kit (Macherey-Nagel, Germany), following the protocol in combination with TRIzol® lysis with small and large RNA in one fraction (total RNA). RNA concentration ng/μl and quality RNA Integrity Number RIN was determined Agilent 2100 Bioanalyzer (Santa Clara, CA). RNA extract was stored at −80 °C until use.

### DNA library preparation and sequencing

One μg of genomic DNA from each of the six samples (3 ovaries and 3 hypothalami) was sonicated to produce DNA fragments of about 350 bp. Methyl-binding domain (MBD) enrichment was performed using the MethylMiner™ Methylated DNA Enrichment Kit (Invitrogen, Carlsbad, CA, USA), following manufacture instruction. Sequencing library construction was performed using the TruSeq® Nano Library Preparation Kit (Illumina, San Diego, CA, USA). Libraries were quality checked and quantified on Agilent 2200 TapeStation, High Sensitivity D1000. The six samples were then used for cluster generation and subsequent sequencing on a single lane of Illumina Hiseq 2000 (San Diego, CA) and 100-base paired-end reads were generated.

### RNA library preparation and sequencing

Two μg of total RNA from each of the six samples (3 ovary and 3 hypothalamus) was used for library construction. Libraries were generated using the Illumina TruSeq® RNA Sample Preparation v2 Kit according to manufacturer’s instructions using poly(A) enrichment. Libraries were quality checked and quantified on Agilent 2200 TapeStation, High Sensitivity D1000. The six samples were then used for cluster generation and subsequent sequencing on a single lane of Illumina Hiseq 2000 (San Diego, CA) and 100-base paired-end reads were generated.

### Bioinformatics analysis

After quality assessment with FastQC (35) raw sequence data cleaned of reads containing adapters, unknown or low quality bases using Trimmomatic (38) and subsequently mapped to the goat reference genome (CHIR_1.0 GenBank assembly accession: GCA_000317765.1, ftp://ftp.ncbi.nih.gov/genomes/Capra_hircus) with BWA-SW [38].

ChIPseeqer [39], a computational framework for the identification of high methylated regions, identified as peaks, across the genome, was used for genome-wide detection of methylation peaks according to the following threshold: t 2 -fold_t 1 -readlen 101 -fraglen 250. ChIPseeqer modules were used to annotate methylated regions according to the reference genome CHIR_v1.0 annotation, and explore methylation distribution in genomic regions (promoters, 3′ UTR, exon, intron, intergenic and distal regions). The analysis of the distribution of methylation peaks per chromosome was performed by dividing the number of peaks by the length of each chromosome. The ratio obtained was further normalized dividing it by the total number of reads per organ. Methylation densities in different genome regions were normalized according to their representation within the genome. The CpG islands were scanned by CpG cluster [40] a distance-based algorithm for CpG-island detection. CGIs were defined according to three criteria: length > 200 bp, ≥50% GC content, ≥ 0.6 of CpG observed/expected. Bedtools v2.25.0 intersect was used to calculate the overlap (of at least 90% length/length) between CGIs and methylation peaks.

RNA-Seq raw data were trimmed using Trimmomatic [41]. Sequences were aligned to the goat reference genome version 1.0 using STAR_2.3.0 [42]. Subsequently, HTSeq-count [43] was used to count sequences aligned to each gene. The software package EdgeR of Bioconductor [44] was used to estimate differential expression between hypothalamus and ovary. Pathway analysis on differentially expressed/methylated genes was performed using STRING 10.0 [45].

### Statistical analysis

*P*-values in tables and figures were calculated with a Student’s t-test with False Discovery Rate (FDR) correction, unless otherwise noted. The Jackknifing simulation test based on the random selection of genes was performed using an in-house R script. For every match (4 in total), 100,000 simulations were tested.

## Additional files

Additional file 1: List and position of CGIs found across the goat reference genome. (XLSX 3401 kb)

Additional file 2: Comparison between methylated CGIs per chromosome in hypothalamus and ovary vs the total number of CGIs. (XLSX 9 kb)

Additional file 3: List of DMRs and DMGs between hypothalamus and ovary. For every DMR a *P*-value and a FDR is shown. (XLSX 395 kb)

Additional file 4: List of DEGs between hypothalamus and ovary. Positive value in the LogFC column refers to genes over-expressed in the vary; negative values indicates genes over-expressed in hypothalamus. (XLSX 1257 kb)

Additional file 5: Pathway analysis (Biological process, Molecular function and Cellular component) of shared genes among negative and positive correlation for DMGs and DEGs. (XLSX 23 kb)

## Abbreviations

CGIs: CpG islands
CHI: Chromosome of *Capra hircus*
CpG: C (cytosine) base followed immediately by a G (guanine) base
DEGs: Differentially expressed genes
DMGs: Differentially methylated genes
DMRs: Differentially methylated regions
MBD-seq: Methyl-CpG-binding domain protein enriched genome-wide sequencing.
MRs: Methylated regions
